# Integrative proteogenomic analyses identify plasma proteins that impact the risk of ischemic stroke

**DOI:** 10.1038/s43856-026-01734-z

**Published:** 2026-07-03

**Authors:** Lazaros Belbasis, Adam von Ende, Parag Gajendragadkar, Elsa Valdes-Marquez, Federico Murgia, Cornelia van Duijn, Jemma C. Hopewell

**Affiliations:** Nuffield Department of Population Health, University of Oxford, Oxford, UK

**Keywords:** Stroke, Target identification

## Abstract

**Background:**

Understanding the proteins implicated in the pathogenesis of ischemic stroke is important for elucidating disease mechanisms and informing prevention strategies. In this study, we aim to identify plasma proteins with a potentially causal effect on risk of ischemic stroke by integrating the largest available genetic data sources for plasma proteins, ischemic stroke and its risk factors.

**Methods:**

We use genome-wide association (GWA) statistics to identify *cis* protein quantitative trait loci for over 3,500 proteins across two proteomic platforms. Subsequently, we perform two-sample Mendelian randomization (MR) to assess the potentially causal relationships between plasma proteins and a) risk of ischemic stroke and its subtypes, and b) well-established cardiovascular risk factors. Downstream analyses include Bayesian colocalization, phenome-wide associations and interrogation of biological databases.

**Results:**

We identify 21 proteins with evidence of potentially causal associations with risk of ischemic stroke or its subtypes at 5% false discovery rate, with 16 supported further by colocalization. Four proteins (CEP85, KNG1, MMUT, SPATA20) represent findings not previously implicated in ischemic stroke through MR, or through cognate genes in GWA studies. Integration of evidence from phenome-wide MR, animal models and tissue-specific gene expression highlights agonists of MMUT, CEP85 and GRK5, and inhibitors of F11 and KNG1 as the most promising for further consideration as targets for prevention of ischemic stroke.

**Conclusions:**

Our study provides the most comprehensive data integration to date supporting the identification and causal relevance of plasma proteins for ischemic stroke and implicating a number of potential therapeutic targets.

## Plain language summary

Ischemic stroke occurs when the blood supply to a part of the brain is interrupted. It is a major cause of death and long-term disability worldwide, but we still do not fully understand the underlying biological processes. Proteins circulating in the blood may help explain why some people are at higher risk. They may also point to new treatments. In this study, we use large-scale genetic data from over a million people. We aim to identify specific proteins in the blood that may play a role in ischemic stroke. After examining more than 3500 proteins, we identified 11 proteins relevant to ischemic stroke. After considering additional existing knowledge, 4 of them appear to be the most promising as potential drug targets. In summary, our findings improve our understanding of stroke biology and highlight new opportunities for preventing ischemic stroke.

## Abstract (web summary)

Belbasis et al. integrate large-scale proteomic and genetic data to identify plasma proteins with potential causal effect on risk of ischemic stroke using Mendelian randomization and colocalization. Four proteins (CEP85, KNG1, MMUT, and SPATA20) are, as far as they are aware, newly identified as implicated in the pathogenesis of ischemic stroke.

## Introduction

Stroke is one of the leading contributors to disability and mortality globally^1^, with ischemic stroke comprising approximately 70% of all cerebrovascular events^2^. Ischemic stroke is a heterogeneous clinical condition. Cardioembolic stroke, large artery stroke, and small vessel stroke are the most prevalent subtypes of ischemic stroke, each with distinct aetiologies and risk factors leading to potential implications for prevention and treatment^2^.

Proteins are crucial for understanding disease pathogenesis, and their potential causal role in disease may also have major implications for the identification of drug targets^3^. Technological advances have made it possible to measure thousands of proteins in large population cohorts^4^, and genome-wide association studies (GWAS) have identified numerous protein quantitative trait loci (pQTLs)^5^. These pQTLs can be used as genetic proxies for protein levels within a Mendelian randomization (MR) framework to understand the potential causal role of a protein in a disease^3^.

Two previous proteome-wide MR studies identified ten plasma proteins (ABO, LPA, TNFSF12, F11, MMP12, SCARA5, CD40, TFPI, TMPRSS5, and CD6) related to ischemic stroke^6,7^. However, the emergence of large-scale cohorts measuring thousands of proteins on diverse platforms and the expansion of GWAS data for both proteins and ischemic stroke, as well as more detailed bioinformatics resources, motivate a more comprehensive approach to identify proteins involved in the pathogenesis of ischemic stroke and to highlight proteins that may be suitable as drug targets for the prevention of ischemic stroke.

In the present study, we aim to (1) identify plasma proteins associated with risk of ischemic stroke and its subtypes using a two-sample MR and colocalization framework, (2) examine the effect of stroke-associated proteins on well-established cardiovascular risk factors, and (3) assess the potential relevance of stroke-associated proteins as drug targets using a comprehensive range of bioinformatics resources.

Our genetic study implicates seven proteins measured on the Olink platform (GRK5, F11, MMP12, FURIN, MMUT, CEP85, PROCR) and five proteins measured on the SomaScan platform (F11, MMP12, KNG1, SH3BGRL3, LRP4) that have potentially causal relevance for ischemic stroke. In subtype-specific analyses, we also show that F11 is implicated in the risk of cardioembolic stroke, MMP12 in the risk of large artery stroke, and SPATA20 in the risk of small vessel stroke, supported by MR and colocalization. After integrating evidence from phenome-wide MR, animal models, and tissue-specific gene expression, we highlight agonists of MMUT, CEP85, and GRK5, and inhibitors of F11 and KNG1 as the most promising targets for the prevention of ischemic stroke.

## Methods

### Data sources

#### GWAS of the human plasma proteome

Genome-wide association statistics were publicly available from two large proteogenomic studies in populations of European ancestry (Supplementary Data 1)^8,9^. In summary, we used data from previously conducted GWAS for circulating protein levels, including: (1) 35,571 UK Biobank participants based on the Olink Explore 3072 platform, which measured 2941 protein analytes representing 2923 unique proteins^8^, and (2) 35,559 participants from the Icelandic Cancer Project and deCODE Health Study based on the SomaScan version 4 platform, which measured 4907 aptamers representing 4719 proteins^9^.

#### GWAS of ischemic stroke and its subtypes

Genome-wide association statistics were publicly available from the GIGASTROKE Consortium. We considered summary statistics from European ancestry cohorts examining total ischemic stroke and ischemic stroke subtypes (i.e., cardioembolic stroke, large artery stroke, and small vessel disease) (Supplementary Data 1)^10^. The sample overlap between these data and the two proteogenomic studies ranges from 0 to 3%.

#### Selection of instrumental variables and data harmonization

Autosomal *cis* pQTLs, which have a direct regulatory effect on the protein expression and proven of greater relevance for downstream translation^5^, were identified in the two proteogenomic studies^8,9^. This approach aimed to limit the potential for horizontal pleiotropy that may occur with the use of *trans* pQTLs. The *cis* region was defined as a distance of 1 Mb upstream or downstream from the end or start, respectively, of the gene encoding the protein of interest in both platforms.

*cis* pQTLs were selected based on the statistical criteria applied in the contributing proteogenomic studies (*p* < 1.70 × 10^−11^ for the study using the Olink platform, and *p* < 1.80 × 10^−9^ for the study using the SomaScan platform)^8,9^. The thresholds for variant selection differ to reflect those of the original studies. Specifically, associations in the Olink study were defined as statistically significant based on adjusting the genome-wide significant threshold for the number of proteins tested^8^, whilst associations in the SomaScan study were defined as statistically significant based on adjusting the nominal significance threshold for the number of genetic variants tested per protein^9^. Ambiguous palindromic single nucleotide polymorphisms with an allele frequency between 0.42 and 0.58 (in either the proteogenomic or ischemic stroke GWAS) were excluded^11^.

Due to the complex linkage disequilibrium (LD) structure of genetic variants within the human major histocompatibility complex (*MHC*) region and their known highly pleiotropic effects, genetic variants and proteins encoded by genes within the *MHC* region (chr6: from 26 Mb to 36 Mb) were excluded^3^. To reduce the risk of weak instrument bias, genetic instruments with an *F*-statistic of at least 10 were included^12^. After restricting genetic variants to those available in GIGASTROKE, clumping based on LD from 1000 Genomes Project European ancestry population using a genetic window of ±10 Mb and an LD *r*^2^ < 0.0001 was undertaken to identify independent *cis* pQTLs for plasma protein abundance. To ensure that the genetic instruments reflect causal effects from the protein to the ischemic stroke phenotypes, we applied the Steiger filtering, which tests whether each genetic instrument explains more variance in the exposure than in the ischemic stroke phenotype^13^. PLINK version 1.90 and the *ieugwasr* R package were used to perform the clumping^14^. We followed the recommended harmonization framework for two-sample MR analyses^11,15^, using the *TwoSampleMR* R package^13^. The selection process for genetic instruments is summarized in Supplementary Fig. S1.

If a protein was targeted by multiple assays within the same proteomic platform, only the assay that had the largest explained variability by the independent instrumental variables was considered to maximize statistical power for MR analysis. There is substantial heterogeneity between the complementary protein information provided by the Olink and SomaScan platforms, given their technological approaches, which can result in different proteoforms of the same protein that are not directly comparable^16^. Consequently, when a protein was measured in both the Olink and SomaScan platforms, we selected genetic instruments separately from each platform and performed MR analyses independently.

### Statistical analysis

#### Association of plasma proteins with stroke and stroke subtypes

The Wald ratio, defined as the ratio of the gene-outcome effect divided by the gene-exposure effect, was calculated for each of the genetic variants selected^17^. When more than one variant was available for a protein, the Wald ratio estimates were combined using a fixed-effect inverse variance weighted model, based on the assumption that *cis* variants act through the same molecular pathway. Statistically significant associations were declared based on a 5% false discovery rate (FDR) threshold (after applying the Benjamini-Hochberg correction)^18^. MR analyses were performed using the *TwoSampleMR* R package^13^.

For proteins quantified on both platforms, we assessed whether evidence of causal effects was consistent across platforms. However, genetic instruments and resulting causal effect estimates from Olink and SomaScan platforms are not directly comparable, given differences in the underlying protein information captured by these platforms and the use of normalized expression (NPX units) and protein abundance (standard deviation units), respectively.

#### Bi-directional (reverse) Mendelian randomization

The potential effect of genetic liability to ischemic stroke on plasma proteins was considered using a bi-directional (reverse) MR approach. To obtain independent genetic variants associated with any ischemic stroke and ischemic stroke subtypes, we performed LD clumping as previously described above, with a statistical significance threshold of *p* < 5 × 10^−8^. We then examined whether genetically predicted risk of any of the ischemic stroke phenotypes was related to the proteins considered. The Wald ratio was estimated for each genetic variant and combined using a random-effects model^19^, to allow for heterogeneity that may result from different genetic variants for ischemic stroke and its subtypes acting through different molecular pathways. Statistically significant associations were determined using a 5% FDR.

#### Bayesian colocalization

Evidence of colocalization is used to support the validity of the genetic variants and strengthen MR findings by reducing the risk of confounding due to LD^20^. In the context of MR, colocalization provides evidence that the genetic variant used as an instrument influences the outcome through the exposure of interest. We examined whether the prioritized proteins share the genetic variant with the outcomes of interest by conducting a colocalization analysis, under the assumption of a single causal variant in each genetic locus. The colocalization analysis was performed on a genetic window of ±0.5 Mb from the lead *cis* pQTL of each protein. We implemented this analysis using the *coloc* R package with its default prior probabilities^21^.

A posterior probability greater than 80% was considered as strong evidence for colocalization, and a posterior probability between 60 and 80% was considered moderate evidence. Proteins with a statistically significant association with any ischemic stroke or its subtypes at 5% FDR, which were also supported by strong evidence for colocalization, were prioritized for subsequent analyses.

#### Pleiotropy-robust *cis* Mendelian randomization

As a sensitivity analysis, we applied the MR-link-2 method to proteins that showed evidence of a causal effect in the primary analysis^22,23^. MR-link-2 is a pleiotropy-robust *cis* MR method that enables causal inference in the presence of LD and unobserved pleiotropic effects, without requiring the removal of pleiotropic genetic instruments or explicit measurement of all sources of pleiotropy. Using this approach, we evaluated the robustness of our findings to potential horizontal pleiotropy (i.e., violation of the exclusion restriction assumption). MR-link-2 tests two parameters: the causal effect of a protein on the outcome conditional on pleiotropic effects, and a pleiotropy parameter that captures the heritable component of the outcome within the *cis* protein locus that is independent of the protein. For these analyses, we used summary statistics for each protein and stroke outcome across the corresponding *cis* region, together with the LD reference panel from the 1000 Genomes Project European ancestry population.

#### Comparison with prior evidence on proteins related to the risk of ischemic stroke

We examined whether (1) any genetic variant within the genes encoding the prioritized proteins was associated with ischemic stroke or its subtypes in the GWAS Catalog^24^, or (2) the prioritized proteins were reported as statistically significant for any ischemic stroke or its subtypes in a proteome-wide MR study. To compile a comprehensive list of relevant MR studies, a systematic review was conducted in PubMed up to 25th October 2025 using the following search algorithm: (“stroke” OR “cerebrovascular disease” OR “small vessel disease”) AND (“Mendelian randomization” OR “Mendelian randomisation”). MR studies were deemed eligible if they assessed the association of at least 100 proteins using plasma *cis* pQTLs measured through a high-throughput platform.

We did not consider (1) studies examining any stroke as an outcome, because this phenotype conflates ischemic and hemorrhagic stroke, which are two very heterogeneous clinical entities, (2) studies examining plasma proteins with a causal effect on outcomes related to stroke progression, (3) studies that did not use colocalization to validate their findings, and (4) studies that did not use genome-wide association statistics from large genetic consortia. From each eligible study, we extracted information on the GWAS datasets used to derive *cis* pQTLs and their effect on ischemic stroke phenotypes, along with the proteins identified.

#### Association of plasma proteins with ischemic stroke risk factors

We focused on five key risk factors for ischemic stroke (ever smoking^25^, systolic blood pressure^26^, type 2 diabetes mellitus^27^, body mass index^28^, and atrial fibrillation^29^), in line with a previously published study, which suggested a causal role for these factors in the development of ischemic stroke^6^. For each risk factor, we used publicly available GWAS data from European populations to identify the genetic effect of each of the *cis* pQTLs on the ischemic stroke risk factors (Supplementary Data 1), applying the same rigorous steps described above. Statistically significant associations were based on a 5% FDR, after applying the Benjamini-Hochberg correction across the associations between all the proteins with ischemic stroke risk factors.

#### Phenome-wide associations

To consider whether protein abundance could have wider implications than on ischemic stroke, we examined associations of the genetic instruments derived for the prioritized proteins across the phenome. Specifically, the effects of the genetic instruments derived for the prioritized proteins were estimated on 57 blood biomarkers in the UK Biobank and 2462 disease outcomes in FinnGen (release 5, accessed through OpenGWAS), using summary-level genetic data from publicly available GWAS^30^. Statistically significant associations were based on 5% FDR threshold.

### Biological data integration

#### Enrichment analysis

For the proteins associated with ischemic stroke or its subtypes and supported by colocalization, we performed an enrichment analysis in Gene Ontology^31^, Reactome Pathway^32^, and Kyoto Encyclopedia of Genes and Genomes (KEGG) databases^33^, using the *clusterProfiler* R package^34^. Statistically significant enrichment was based on 5% FDR. All proteins tested for an association with ischemic stroke or its subtypes were included in the analysis as the background set^35,36^.

#### Protein-protein interactions and Mendelian diseases

We used the Search Tool for the Retrieval of Interacting Genes/Proteins (STRING) to examine potential protein-protein interactions among proteins with evidence of colocalization with ischemic stroke^37^. We also searched the Online Mendelian Inheritance in Man (OMIM) to assess whether the identified proteins have been linked to any genetic disorders^38^.

#### Assessment of potential pleiotropic effects of *cis* pQTLs

We assessed potential pleiotropic effects of the selected *cis* pQTLs for the statistically significant proteins by examining whether these genetic variants have a genome-wide significant effect on other plasma proteins (*p* < 5 × 10^−8^), by testing the genetic effects of these *cis* pQTLs on all the Olink and SomaScan proteins measured in the UK Biobank and deCODE study, respectively. We then examined whether these associations between *cis* pQTLs and proteins may be explained by mediation through shared biological pathways, which is an indication of vertical pleiotropy, or unrelated mechanisms, which indicates the presence of horizontal pleiotropy. A pleiotropic effect was attributed to mediation if the *cis-* and *trans*-associated proteins were involved in the same biological pathway based on Gene Ontology^39^. Otherwise, we consider them as a potential result of unrelated mechanisms.

#### Feasibility of drug targeting

We searched Open Targets^40,41^, Therapeutic Target Database^42^, Drug Bank^43^, and PharmGKB^44^ to examine whether the prioritized proteins have been previously considered as drug targets in experimental studies in humans or animals. Data on the tractability and do-ability of targeting these proteins were also extracted from Open Targets^45^. A particular protein was considered tractable if (1) it is a membrane or a secreted protein, or (2) it binds a specific ligand or a small molecule, or (3) it has predicted pockets. Targeting a particular protein was considered do-able if (1) at least one mouse gene contains a sequence with a 100% of identity to the target protein, or (2) a high-quality chemical probe is available for targeting this protein. Information about knockout animal models focusing on proteins that are associated with the risk of ischemic stroke was extracted from Open Targets, which curates information based on the Mouse Genome Informatics database^46^.

#### Gene expression

The Human Protein Atlas was used to assess the tissue, brain, and single-cell specificity of the identified proteins^47,48^. We specifically examined if the prioritized proteins show high specificity in tissues or single cells that are relevant to ischemic stroke and its well-established risk factors (i.e., kidney, liver, pancreas, adipose tissue, cardiovascular system, and brain).

### Ethics approval

In this study, we solely used publicly available summary-level GWAS data. Details on how to access these data sets are provided in the “Data availability” statement. For all included GWAS, all participants provided informed consent, and study protocols were approved by their respective ethical committees. As our analyses did not involve individual-level participant data, no additional ethical approval was required for the present study.

## Results

### Association of genetically predicted proteins with ischemic stroke

**Fig. 1:**
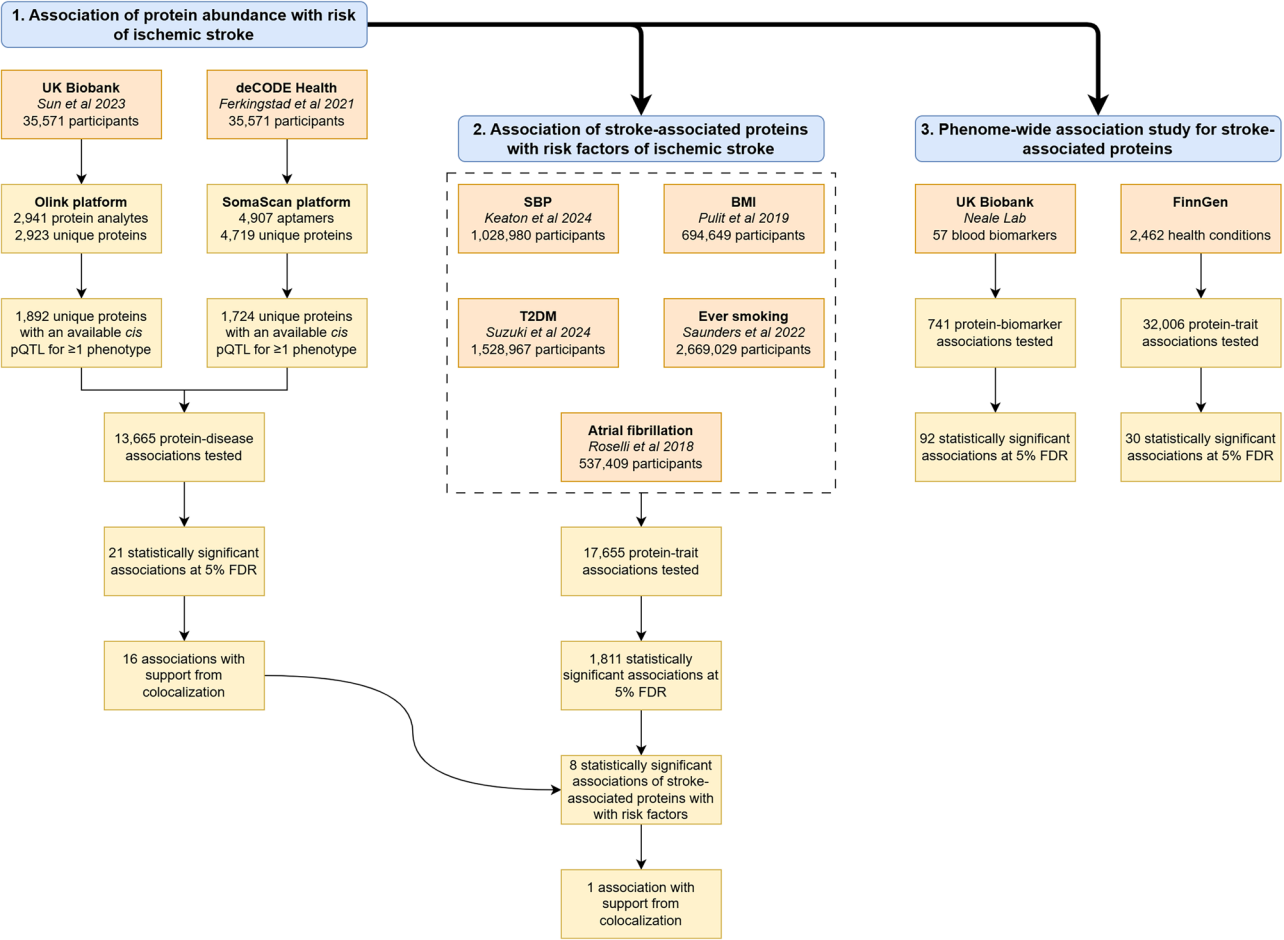
Flow chart of the study design and main results. In the first step, *cis* pQTLs from the UK Biobank (Olink platform) and the deCODE Health Study (SomaScan platform) were used to identify plasma proteins related to ischemic stroke and its subtypes. Through a two-sample Mendelian randomization approach, we identified 21 statistically significant protein-disease associations, and 16 of these protein-disease pairs also showed evidence of colocalization. In the second step, we identified 8 out of 16 statistically significant associations between plasma proteins and ischemic stroke risk factors, and one protein-risk factor pair also showed evidence of colocalization. In the final step, a phenome-wide Mendelian randomization analysis revealed 92 associations with medical conditions in FinnGen and 30 associations with blood biomarkers in the UK Biobank.

**Fig. 2:**
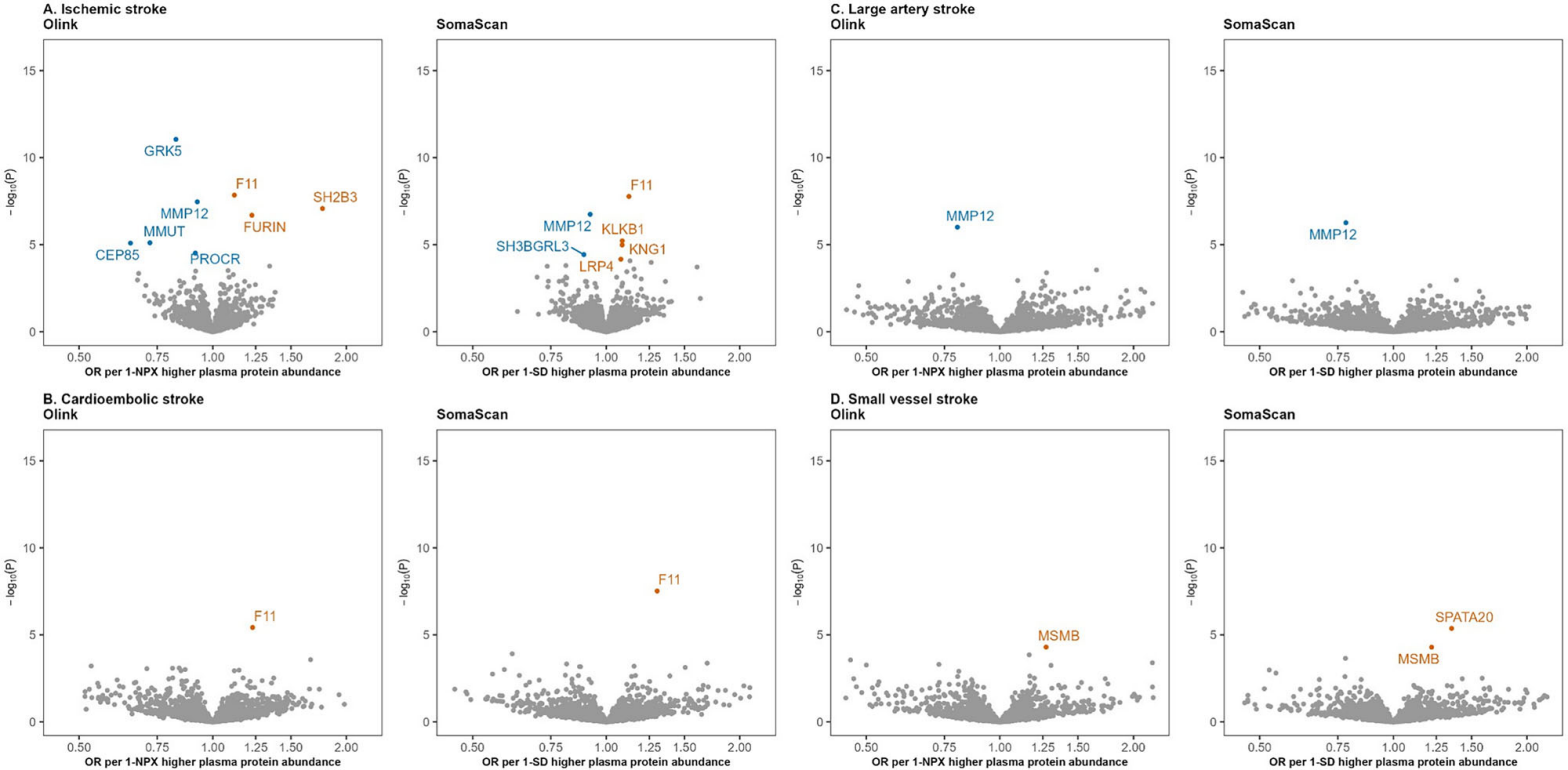
Association of plasma proteins with risk of ischemic stroke and its subtypes using the *cis* two-sample Mendelian randomization approach. Proteins that showed a statistically significant association with ischemic stroke phenotypes, based on 5% FDR, are annotated. The labeled proteins in blue indicate a negative association with stroke risk, while those in orange indicate a positive association. The gray dots represent proteins that did not reach statistical significance. Results are shown separately for each ischemic stroke phenotype: **A** any ischemic stroke, **B** cardioembolic stroke, **C** large artery stroke, and **D** small vessel stroke.

**Fig. 3:**
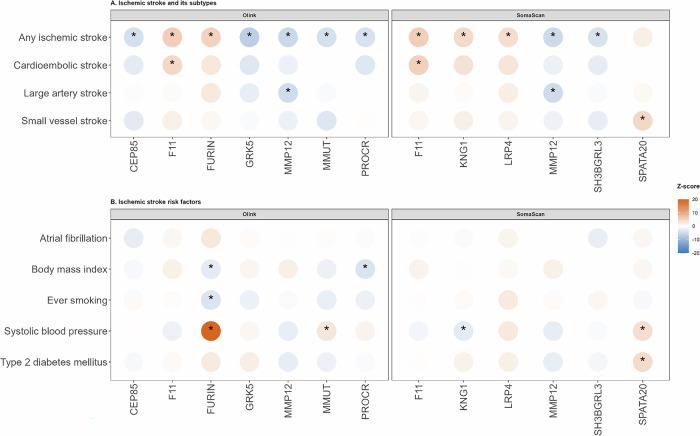
Association of ischemic stroke-associated proteins with ischemic stroke risk factors using two-sample Mendelian randomization analysis and *cis* pQTLs as instrumental variables. Only plasma proteins showing an association with ischemic stroke and/or its subtypes, with support from the two-sample *cis* MR analysis and colocalization, are shown in this figure. **A** illustrates associations with ischemic stroke and its subtypes (including cardioembolic, large artery, and small vessel stroke). **B** shows associations with ischemic stroke risk factors (atrial fibrillation, body mass index, smoking status, systolic blood pressure, and type 2 diabetes). Positive associations (orange) and negative associations (blue) are represented by the color intensity, scaled by the *Z*-score. Asterisks indicate significant associations at 5% FDR.

To systematically evaluate the evidence for a potentially causal association between plasma proteins and risk of ischemic stroke and ischemic stroke subtypes, we performed a proteome-wide two-sample MR analysis (Fig. 1). We used independent *cis* pQTLs from two complementary proteomic platforms (Olink in the UK Biobank and SomaScan in the deCODE Health Study) as genetic proxies for plasma protein abundance. In total, we tested 13,665 associations (53% using pQTLs from the Olink platform and 47% using pQTLs from the SomaScan platform). We observed 1054 (7.7%) nominally significant associations (*p* < 0.05; Supplementary Data 2), and 21 associations (0.2%) were statistically significant at a 5% false discovery rate (FDR), corresponding to *p* < 6.76 × 10^−5^ (Figs. 2 and 3 and Table 1). Steiger filtering did not exclude any genetic instruments and validated that the genetic instruments for all the tested associations reflect causal effects from the protein to ischemic stroke phenotypes^13^. None of the genetic variants used in these 21 associations was protein-altering, indicating the robustness of our analysis against epitope binding effects^49^.

**Table 1:** Plasma proteins with a statistically significant effect on ischemic stroke phenotypes at 5% FDR using two-sample Mendelian randomization

Platform	Assay ID	Protein	N variants^a^	OR^b^	95% CI	*p*^c^	Novel^d^	Colocalization^e^
	Ischemic stroke							
Olink	OID20066	GRK5	1	0.83	0.78–0.87	8.82E−12	No	0.99
	OID30773	F11	2	1.12	1.08–1.16	1.41E−08	No	0.99
	OID21439	MMP12	3	0.92	0.90–0.95	3.43E−08	No	0.90
	OID21222	SH2B3	1	1.77	1.44–2.18	8.30E−08	NA	0.59
	OID21514	FURIN	2	1.23	1.13–1.32	2.02E−07	No	0.86
	OID30159	MMUT	1	0.72	0.63–0.83	7.71E−06	Yes	0.81
	OID21241	CEP85	1	0.65	0.54–0.79	8.03E−06	Yes	0.86
	OID30149	PROCR	1	0.91	0.88–0.95	3.01E−05	No	0.90
SomaScan	2190_55	F11	3	1.12	1.08–1.17	1.68E−08	No	0.99
	4496_60	MMP12	3	0.92	0.89–0.95	1.79E−07	No	0.84
	4152_58	KLKB1	3	1.09	1.05–1.13	5.92E−06	NA	0.14
	15343_337	KNG1	3	1.09	1.05–1.13	1.03E−05	Yes	0.99
	17490_4	SH3BGRL3	2	0.89	0.84–0.94	3.68E−05	No	0.84
	19558_10	LRP4	2	1.08	1.04–1.12	6.67E−05	No	0.86
	Cardioembolic stroke							
Olink	OID30773	F11	2	1.23	1.13–1.34	3.76E−06	No	0.99
SomaScan	2190_55	F11	3	1.30	1.19–1.43	3.01E−08	No	0.99
	Large artery stroke							
Olink	OID21439	MMP12	3	0.80	0.73–0.88	9.78E−07	No	0.77
SomaScan	4496_60	MMP12	3	0.78	0.71–0.86	5.41E−07	No	0.91
	Small vessel stroke							
Olink	OID20275	MSMB	2	1.27	1.13–1.43	5.01E−05	NA	0.52
SomaScan	11117_2	SPATA20	2	1.35	1.19–1.54	4.22E−06	Yes	0.92
	10620_21	MSMB	2	1.22	1.11–1.34	5.07E−05	NA	0.46

*CI* confidence interval, *OR* odds ratio,* NA* not assessed.

^a^For proteins with a single genetic variant as instrumental variable, odds ratio was estimated using the Wald ratio. For proteins with at least 2 genetic variants as instrumental variables, odds ratio was estimated using the fixed-effect inverse variance-weighted model.

^b^The odds ratio represents the risk of stroke by 1 standard deviation increase in plasma protein abundance for *SomaScan* platform, and by 1 NPX unit increase in plasma protein abundance for *Olink* platform.

^c^*p* values are two-sided and are based on *Z* tests. Multiple testing was addressed using the Benjamini-Hochberg false discovery rate procedure, although the table presents the nominal (unadjusted) *p* values.

^d^Novelty was defined based on the findings of the latest genome-wide association study and previously published Mendelian randomization studies, and it was assessed only when posterior probability for colocalization exceeded 80%.

^e^Colocalization was performed under the assumption of a single causal variant per genetic loci. This column reports the posterior probability that each protein shares the same causal variant with an ischemic stroke phenotype at the respective genetic loci.

Higher genetically-predicted levels of five proteins (F11, FURIN, KNG1, LRP4, SH2B3) were associated with higher risk of any ischemic stroke, with odds ratio ranging from 1.08 (95% CI, 1.04–1.12) per 1-SD higher LRP4 abundance to 1.77 (95% 1.44–2.18) per 1-NPX higher SH2B3 abundance. Also, higher levels of six proteins (GRK5, MMP12, MMUT, CEP85, PROCR, SH3BGRL3) were associated with lower risk of any ischemic stroke with odds ratio ranging from 0.65 (95% CI, 0.54–0.79) to 0.92 (95% 0.90–0.95) per 1-NPX higher CEP85 and MMP12 abundance, respectively. Furthermore, the effects of these proteins on risk of ischemic stroke were directionally consistent in the GIGASTROKE multi-ancestry population (~30% non-Europeans; Supplementary Data 3), based on the same *cis* pQTLs.

Stroke subtype-specific associations were also considered, showing higher genetically-predicted MMP12 abundance associated with lower risk of large artery stroke (OR per 1-SD higher MMP12, 0.78; 95% CI, 0.71–0.86), and higher F11 abundance with risk of cardioembolic stroke (OR per 1-SD higher, 1.30; 95% CI, 1.19–1.43). Notably, higher MSMB abundance (OR per 1-SD higher, 1.22; 95% CI, 1.11–1.34) and higher SPATA20 abundance (OR per 1-SD higher, 1.35; 95% CI, 1.19–1.54) were associated exclusively with small vessel stroke.

### Colocalization of plasma proteins with ischemic stroke

Assuming a single causal genetic variant at each genetic locus, we found that 16 out of 21 statistically significant associations (76%) had strong evidence for colocalization (posterior probability for H_4_ > 0.80; Supplementary Data 4). One additional protein-disease pair (MMP12 measured through Olink platform with large artery stroke) showed moderate evidence for colocalization with large artery stroke (posterior probability for H_4_ = 0.77). Two associations (SH2B3 and KLKB1 with any ischemic stroke) suggested a distinct causal variant (posterior probability for H_3_ > 0.80). In summary, the colocalization analysis provides an additional level of support for these associations, strengthening the validity of the *cis* pQTLs as genetic instruments for these proteins^20^.

### Pleiotropy-robust *cis* Mendelian randomization

Results were broadly consistent in the sensitivity analysis using MR-link-2, a pleiotropy-robust *cis* MR method^22^. Of the 21 associations detected in our primary analysis, 19 (90%) remained statistically significant after adjusting for pleiotropy (*p* < 0.05; Supplementary Data 5). For SH2B3, the pleiotropy-adjusted effect on risk of ischemic stroke was not statistically significant, with the MR-link-2 pleiotropy test indicating substantial horizontal pleiotropy. This is consistent with the lack of colocalization between SH2B3 and ischemic stroke at the *cis* locus, as we previously reported. For MMUT, the pleiotropy-adjusted effect estimate was directionally consistent with the primary analysis but was not statistically significant. This finding may reflect a combination of limited statistical power due to low heritability for MMUT at the *cis* region and presence of horizontal pleiotropy.

### Cross-platform comparison

We compared the plasma proteins we identified as potentially causal for ischemic stroke and its subtypes across the two different proteomic platforms (Supplementary Fig. S2 and Supplementary Data 6). Five associations (F11 with ischemic stroke and cardioembolic stroke, MMP12 with ischemic stroke and large artery stroke, and MSMB with small vessel disease) were statistically significant in both platforms with concordant direction of effect, whereas one association (KLKB1 with any ischemic stroke) was statistically significant only when we considered *cis* pQTLs from the SomaScan platform. The remaining associations could be evaluated only in one platform due to the limited overlap in the proteins measured across Olink and SomaScan platforms. A directionally consistent effect across the two platforms indicates that the inferred effect on disease risk is independent of the platform used for protein abundance measurement and pQTL identification.

### Effect of genetic liability to ischemic stroke on plasma proteins

To assess the relationship of genetic liability to ischemic stroke or its subtypes with plasma proteins, we conducted a reverse MR analysis using genetic proxies for risk of ischemic stroke or its subtypes. Associations between genetic liability to small vessel stroke and the plasma proteome could not be assessed due to absence of genome-wide significant genetic variants for European populations in the GWAS by the GIGASTROKE Consortium^10^.

Among 11,238 associations tested (Supplementary Data 7), 827 were statistically significant at *p* < 0.05, and 325 were statistically significant at 5% FDR. However, none of these proteins overlapped with those found to have statistically significant effects on risk of ischemic stroke and its subtypes. This observation supports that the identified plasma proteins are causally relevant to, rather than a consequence of, ischemic stroke.

### Comparison of our findings with existing studies

Our systematic review identified eight proteome-wide MR studies (six in European populations, one in East Asian populations, and one in South Asian populations) examining the association of proteins with risk of ischemic stroke and/or its subtypes (Table 2)^6,50–56^. Seven of the proteins we identified (GRK5, PROCR, F11, MMP12, SH3BGRL3, LRP4, and FURIN) had been previously observed in MR studies and effects were directionally consistent, thus our results support these previous findings. There was also genetic support from the GIGASTROKE Consortium, which identified genetic variants in five of the 11 genes (*GRK5*, *F11*, *PROCR*, *FURIN*, and *MMP12*) encoding proteins that are associated with ischemic stroke in populations of European ancestry^10^. To the best of our knowledge, our study supports MR and GWAS findings for seven proteins, and identifies four proteins that have not previously been implicated in risk of ischemic stroke: MMUT, CEP85, and KNG1 associated with ischemic stroke, and SPATA20 with small vessel stroke. Also, by examining the data sources for ischemic stroke and pQTL selection used in the previous studies, our study offers a unique combination of two large GWAS on plasma proteins using two different proteomic platforms with the largest GWAS for ischemic stroke in populations of European ancestry.

**Table 2:** Studies examining the effect of plasma proteins on ischemic stroke and its subtypes using protein quantitative trait loci for at least 100 proteins within a Mendelian randomization and colocalization framework

First author (Year)	Ancestry	Stroke GWAS	Source of pQTLs	Proteins identified
Chen (2022)^6^	Europeans	MEGASTROKE^97^	INTERVAL (Olink)^98^	Ischemic stroke: CD40, TFPI, MMP12, IL6RA, TMPRSS5; Large artery stroke: CD40, MMP12; Cardioembolic stroke: TMPRSS5
Wu (2022)^50^	Europeans	MEGASTROKE^97^	AGES Reykjavik study (SomaScan)^99^	Ischemic stroke: ALDH2, PTPN11, VPS36, SLC44A2 Cardioembolic stroke: L3HYPDH; Small vessel stroke: ICA1L
Wu (2024)^51^	South Asians	GIGASTROKE^10^	UK Biobank (Olink)^8^	Cardioembolic stroke: GP6
Yao (2025)^52^	Europeans, East Asians	GIGASTROKE^10^	UK Biobank (Olink)^8^, China Kadoorie Biobank (Olink)	Ischemic stroke: FGF5, TMPRSS5, FURIN, F11, ALDH2, ABO, GRK5, KIAA0319, PROCR, MMP12
Zhang (2025)^53^	Europeans	MEGASTROKE^97^	INTERVAL (Olink)^98^	Ischemic stroke: MMP12
Zhao (2025)^54^	Europeans	MEGASTROKE^97^	UK Biobank (Olink)^8^, deCODE (SomaScan)^9^	Ischemic stroke: FURIN; Cardioembolic stroke: F11; Small vessel stroke: VSIR, DDHD2
Zhu (2024)^55^	Europeans	GIGASTROKE^10^	deCODE (SomaScan)^9^, ARIC (SomaScan)^100^	Ischemic stroke: F11, LRP4, MMP12, PTGR1, SH3GBRL3; Large artery stroke: MMP12; Cardioembolic stroke: F11, EMILIN3, NUDT9
Zou (2024)^56^	Europeans	MEGASTROKE^97^, FinnGen, UK Biobank	KORA F4/QMDiab (SomaScan)^101^, INTERVAL (Olink)^98^, Framingham Heart study (Luminex)^102^, IMPROVE (Olink)^103^, deCODE (SomaScan)^9^	Ischemic stroke: SWAP70, MMP12; KLKB1

The table summarizes proteome-wide Mendelian randomization studies evaluating the associations of genetically predicted plasma proteins with risk of ischemic stroke or its subtypes. For each study, we present the genetic ancestry of the study population, the genetic data sources for ischemic stroke and for identification of protein quantitative trait loci (pQTLs), and the proteins prioritized. Studies were identified through a systematic review, as described in the “Methods”.

### Pleiotropic effects of *cis* pQTLs

Genetic variants used as instruments for a plasma protein may exhibit effects on other proteins, which may be attributed to either shared biological pathways (*vertical pleiotropy*) or unrelated mechanisms (*horizontal pleiotropy*). These effects were explored using predicted protein–protein interactions in STRING and biological pathway annotations in Gene Ontology (Supplementary Figs. S3 and S4 and Supplementary Data 8 and 9).

**Fig. 4:**
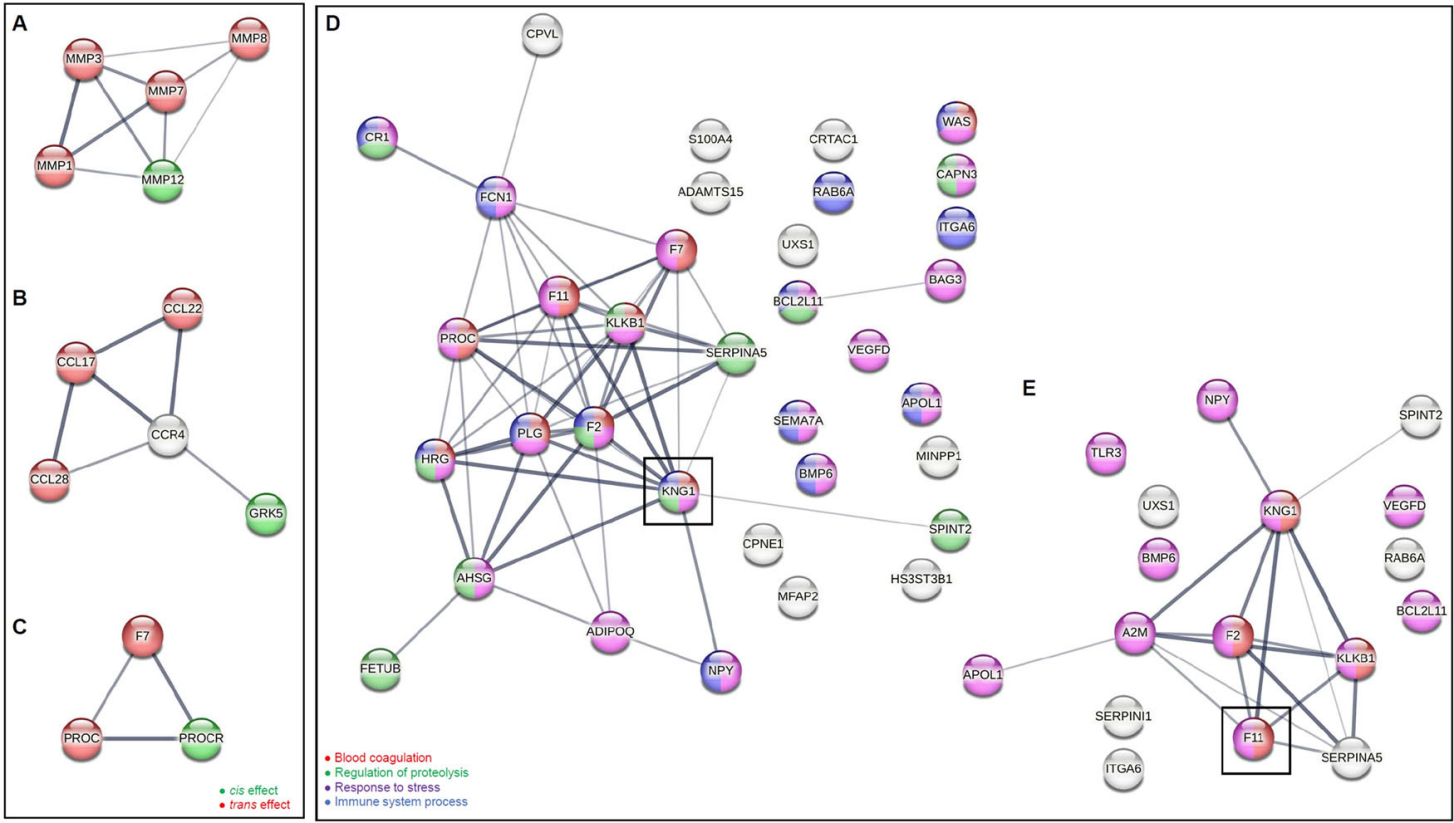
Predicted protein–protein interaction networks for pleiotropic effects of the genetic instruments. Protein–protein interaction networks were generated using the STRING database (version 12). Each node represents a protein, and edges indicate predicted interactions based on evidence from curated databases, experimental data, co-expression, and text mining. Edge thickness reflects the strength of the supporting evidence. In **A**, the *cis* pQTL for MMP12 had an effect on MMP1, MMP3, MMP7 and MMP8, which form an interconnected network in STRING and are collectively involved in collagen degradation according to Gene Ontology (GO). In **B**, the *cis* pQTL for GRK5 was associated with CCL28, CCL17 and CCL22, which participate in the G protein-coupled receptors signaling based on GO. When an additional protein (CCR4) was introduced, all these proteins form a cohesive interaction network. In **C**, the *cis* pQTL had an effect on PROC and F7, which form an interaction network based on STRING and participate in the formation of fibrin clot based on GO. In **D**, a complex protein–protein interaction network was observed for the proteins that are affected by the *cis* pQTLs for KNG1, with most of the proteins relating to four particular biological processes: blood coagulation, regulation of proteolysis, response to stress and immune system process. In **E**, the complex protein–protein interaction network for the proteins that are affected by the *cis* pQTLs for F11 is shown. A majority of these effects can be explained by two biological processes in Gene Ontology, which are blood coagulation, and response to stress.

As might be anticipated, many pleiotropic effects were consistent with mediation through shared biological pathways. The *cis* pQTLs for MMP12 were associated with at least one of the matrix metalloproteinase family members (MMP1, MMP3, MMP7, MMP8), all involved in collagen degradation (Fig. 4A). The *cis* pQTL of GRK5 (rs10886430) was associated with CCL17, CCL22, and CCL28, all involved in the G protein-coupled receptors signaling (Fig. 4B). Similarly, the *cis* pQTL for FURIN (rs2071410) was associated with FER and FES, which are involved in the receptor tyrosine kinases signaling. Additionally, the *cis* pQTL for PROCR (rs146102159) was associated with F7 and PROC, all involved in the formation of fibrin clots, indicating possible mediation (vertical pleiotropy, Fig. 4C). However, its association with CPNE1 could not be explained by shared biological pathways, suggesting possible confounding by unrelated mechanisms.

More complex scenarios were observed in the case of KNG1 and F11, as their *cis* pQTLs were associated with multiple proteins. Shared biological mechanisms, related to blood coagulation, proteolysis, immune system, and response to stress, explain the majority of these pleiotropic effects (Fig. 4D, E). Interestingly, the *cis* pQTLs for KNG1 were associated with F11, and vice versa, suggesting a biological interaction between these proteins. Moreover, the *cis* pQTLs for SH3BGRL3, CEP85, LRP4, MMUT and SPATA20 showed minimal pleiotropic effects being associated with one or two additional proteins. Although no shared biological pathways could be identified, this may reflect limitations in the current biological knowledge as captured by the existing databases rather than horizontal pleiotropy.

### Effect of plasma proteins on ischemic stroke risk factors

To assess whether genetically-predicted ischemic stroke-associated proteins have an effect on established risk factors of ischemic stroke^6^, we performed a proteome-wide two-sample MR analysis, using ischemic stroke risk factors as the outcome. Among 17,655 tested associations between plasma proteins and risk factors, 1811 (10.2%) were statistically significant at 5% FDR (Supplementary Data 10). Only eight associations involved ischemic stroke-associated proteins (Fig. 3).

Genetically-predicted FURIN was associated, with systolic blood pressure (*β* per 1-NPX unit higher, 2.71 mmHg; 95% CI, 2.44–2.99), ever smoking (OR per 1-NPX unit higher, 0.96; 95% CI, 0.95–0.98) and BMI (*β* per 1-NPX unit higher, −0.02 SD; 95% CI, −0.04 to −0.01). Genetically-predicted MMUT was positively associated with systolic blood pressure (*β* per 1-NPX unit higher, 0.71 mmHg; 95% CI, 0.23–1.19). Genetically-predicted SPATA20 was associated with type 2 diabetes mellitus (OR per 1-SD increase, 1.05; 95% CI, 1.03–1.08) and systolic blood pressure (*β* per 1-SD higher, 0.30 mmHg; 95% CI, 0.15–0.45). Genetically-predicted KNG1 was associated with SBP (*β* per 1-SD higher, −0.18 mmHg; 95% CI, −0.31 to −0.05). Finally, genetically-predicted levels of PROCR were associated with body mass index (*β* per 1-NPX unit higher, −0.02 SD; 95% CI, −0.04 to −0.01). Only the association of FURIN with systolic blood pressure showed strong evidence of colocalization (posterior probability = 0.98; Supplementary Data 11).

### Assessment of phenome-wide effects

To assess whether changes in the abundance of the ischemic stroke-associated proteins have wider implications by affecting the risk of other medical conditions or the levels of blood biomarkers, we examined the phenome-wide effects of these proteins through a two-sample phenome-wide MR analysis.

We tested the potentially causal association of plasma proteins with multiple diseases in the population of FinnGen. We identified 30 statistically significant protein-disease associations at 5% FDR, involving GRK5, FURIN, MMP12, F11, and PROCR (Supplementary Data 12 and Supplementary Fig. S5). Higher genetically-predicted abundance of F11 and PROCR were associated with higher risks of venous thromboembolism and its subtypes (pulmonary embolism or deep venous thrombosis), despite PROCR being associated with lower risk of ischemic stroke. In addition, higher genetically-predicted abundance of FURIN was associated with higher risks of hypertensive disorders, GRK5 with higher risk of ischemic stroke and its subtypes, and MMP12 with higher risk of allergic asthma.

We further tested the association of these proteins with multiple blood biomarkers in the population of UK Biobank. We found that 92 protein-biomarker associations were statistically significant at 5% FDR, involving all but two proteins (MMP12 and PROCR) (Supplementary Data 13 and Supplementary Fig. S6). We observed associations between ischemic stroke-associated proteins and circulating lipids (CEP85, FURIN, LRP4 and SPATA20), platelet markers (CEP85, FURIN, GRK5, F11, LRP4 and SH3BGRL3), markers of liver (FURIN, LRP4, MMUT and SPATA20) and kidney function (SH3BGRL3, FURIN and SPATA20), and glycemic traits (LRP4 and MMUT). These findings suggest potential biological pathways through which these proteins may influence the risk of ischemic stroke, whereas they may also indicate potential safety considerations if these proteins were pharmacologically targeted.

### Enrichment analysis

Enrichment analysis for the 11 ischemic stroke-associated proteins revealed statistically significant enrichment for complement and coagulation cascades in KEGG pathways, and for formation of fibrin clots in Reactome Pathways, at 5% FDR. Moreover, an interaction of KNG1 with both F11 and GRK5 was observed based in the STRING database. The interaction between F11 and KNG1 was further supported by our analysis, which showed that *cis* pQTLs for F11 was a *trans* pQTL for KNG1 levels, and vice versa (Supplementary Figs. S2 and S3).

### Integration of existing biological evidence

We integrated data from multiple publicly available databases to evaluate the therapeutic potential of the candidate proteins. Three proteins (F11, FURIN and MMP12) have already been investigated in clinical trials with either approved or experimental inhibitors of these proteins (Table 3). Using data from the Open Targets platform, we further assessed the feasibility of targeting the proteins of interest (Table 3). Nine proteins were identified as potentially tractable, meaning that either they function as membrane or secreted proteins, bind specific ligands or small molecules, or they possess predicted binding pockets. Six proteins were highlighted as potentially promising drug targets, supported by the presence of identical gene sequences in mice or the availability of high-quality chemical probes.

**Table 3:** Cumulative evidence on the potential drug targets for ischemic stroke

Protein	Direction of effect^a^	Available drug^b^	Tractability^c^	Doability^d^	Phenome-wide effects^e^	Mouse models	Tissue specificity^f^	Single-cell specificity^f^	Previously reported^g^
CEP85	↓	No	○	○	Platelets	○	○	●	●
F11	↑	Inhibitors	●	○	Lymphocytes, platelets	○	●	●	○
FURIN	↑	No	●	●	Calcium, Albumin, WBC, RBC, LDL cholesterol, cystatin C, HDL cholesterol	●	●	●	○
GRK5	↓	No	●	○	Platelets, Lymphocytes	○	○	●	○
KNG1	↑	Inhibitors	●	○	Not detected	○	●	●	●
LRP4	↑	No	○	○	Calcium, Albumin, triglycerides, platelets	●	●	●	○
MMP12	↓	Inhibitors	●	●	Not detected	○	○	○	○
MMUT	↓	No	●	●	Not detected	○	●	●	●
PROCR	↓	No	●	●	Venous thromboembolism	○	●	○	○
SH3BGRL3	↓	No	●	●	Neutrophils	○	○	○	○
SPATA20	↑	No	●	●	Creatinine	●	○	○	●

^a^The direction of the effect on risk of ischemic stroke for increasing levels of protein abundance based on our two-sample Mendelian randomization.

^b^Based on Open Targets, Drug Bank, Therapeutic Targets Database and PharmGKB.

^c^A particular protein was considered as tractable if (a) it is a membrane or a secreted protein, or (b) it binds a specific ligand or a small molecule, or (c) has predicted pockets.

^d^Targeting a particular protein was considered as do-able if (a) there is at least one gene in mice that contains a sequence with a 100% of identity with the target protein, or (b) there is a high-quality chemical probe targeting this protein.

^e^The information is based on our Mendelian randomization phenome-wide association study in the UK Biobank (for blood-based biomarkers) and FinnGen (for disease phenotypes).

^f^The information is based on Human Protein Atlas and reflects the gene expression specificity in biologically meaningful tissues and single cells for ischemic stroke.

^g^The assessment is based on GWAS Catalog and the published large-scale proteome-wide Mendelian randomization studies for ischemic stroke.

We also used data from the Human Protein Atlas to examine the expression of the genes related to these proteins across multiple tissues and single cells. Six proteins showed high specificity in tissues relevant to ischemic stroke (F11, FURIN, KNG1 and MMUT in liver; LRP4 in brain; and PROCR in adipose tissue), and seven proteins showed high specificity in single cells relevant to ischemic stroke (F11, FURIN, KNG1 and MMUT in hepatocytes; MMUT and CEP85 in cardiomyocytes; LRP4 in oligodendrocytes precursor and astrocytes; and GRK5 in smooth muscle cells and microglial cells). These proteins showed direct biological relevance to ischemic stroke through their highly specific gene expression patterns.

We also reviewed known human genetic disorders from the OMIM database to better understand the biological roles of these proteins. Known genetic disorders were identified for *F11* (deficiency of Factor XI), *KNG1* (kininogen deficiency, and hereditary angioedema), *MMUT* (methylmalonic aciduria), and *LRP4* (myasthenic syndrome, Cenani-Lenz syndactyly syndrome, and sclerosteosis).

We further explored the biological relevance of these proteins to the pathogenesis of ischemic stroke and potential safety considerations using the Mouse Genome Informatics database. Knockout models have revealed that inactivation of *KNG1* and *F11* reduces the risk of thrombotic events. Deletion of *SPATA20* leads to male infertility, while loss-of-function mutations in *FURIN* and *LRP4* are linked to various pathological phenotypes during embryonic development, indicating their potential lack of suitability as drug targets for women of reproductive age. *LRP4* knockout also led to significant abnormalities in motor neuron morphology.

### Cumulative evidence on the potential for drug targeting

By synthesizing evidence from all above-mentioned sources (Table 3), agonists of MMUT, and inhibitors of KNG1 and F11 emerge as promising drug targets. Indeed, MMUT is a promising drug target due to its tractability, doability, high tissue specificity, and potential absence of associations that may represent undesirable effects, whereas loss-of-function mutations in *MMUT* have been linked to ischemic stroke in the context of methylmalonic aciduria, supporting its biological relevance. KNG1 and F11 inhibitors are also promising candidate drugs, with pharmacological inhibitors already available for both proteins, and additional supportive evidence from knockout animal models. However, caution may be needed due to their complex protein interaction network of KNG1 and F11, as we showed earlier (Fig. 4D, E). Similarly, agonists of GRK5 and CEP85 may be suitable targets for prevention of ischemic stroke with phenome-wide MR linking both of them to platelet traits.

The remaining candidate proteins display potential concerns as drug targets. Based on findings from animal models, LRP4 and FURIN inhibitors may lead to abnormalities in embryonic development, raising major safety concerns in women of reproductive age. Similarly, our phenome-wide MR showed that PROCR agonists present a trade-off: they are associated with lower risk of ischemic stroke but higher risk of venous thromboembolism. Meanwhile, the absence of tissue specificity for MMP12, SH3GBRL3 and SPATA20 would de-prioritize them as potential drug targets, given narrow gene expression (i.e., high tissue specificity) is considered desirable for a drug target due to reduced risk of side effects^57^.

## Discussion

After performing a comprehensive assessment of more than 3500 plasma proteins for an association with ischemic stroke and its subtypes, our study provided genetic evidence through MR and colocalization for four proteins (KNG1, MMUT, CEP85, and SPATA20), which have not been previously implicated in the risk of ischemic stroke, and seven previously-identified proteins (GRK5, F11, PROCR, FURIN, LRP4, SH3GBRL3, and MMP12) involved in the pathogenesis of ischemic stroke. These proteins highlight particular physiological pathways for ischemic stroke, including coagulation process, regulation of blood pressure, and energy metabolism.

Our findings underscore the role of the coagulation pathway in the pathogenesis of ischemic stroke by identifying three proteins (F11, KNG1, PROCR) directly involved in coagulation. We found that higher plasma F11 leads to higher risk of ischemic stroke, which is consistent with previous MR studies^6,7^. This finding is further supported by mouse models, where inhibition of F11 protects against acute ischemic stroke^58^. Observational studies suggest that patients with severe F11 deficiency have lower incidence of ischemic stroke^59^, while elevated F11 is associated with higher risk of ischemic stroke^60^. Phase II randomized controlled trials (RCTs) have yielded inconclusive results in terms of efficacy and safety of F11 inhibitors for primary and secondary prevention of ischemic stroke^61^. Moreover, F11 interacts with KNG1, which is cleaved by kallikrein to release the pro-inflammatory mediator bradykinin^62^. Inhibiting KNG1 has been shown to reduce thrombus formation, improve cerebral blood flow, and protect from blood-brain barrier damage in mouse models^63^. *KNG1* genetic locus has been linked to activated partial thromboplastin time further supporting its role in coagulation^64,65^. Additionally, PROCR is involved in coagulation by acting as a receptor for protein C^66^. Preclinical studies have shown that protein C has anti-inflammatory, antithrombotic and neuroprotective properties with potential benefits for protein C as a drug target^67–69^. Protein C was associated with risk of ischemic stroke in the ARIC study^70^. However, we showed that although PROCR agonists protect from risk of ischemic stroke, they are associated with higher risk of deep venous thrombosis and pulmonary embolism, which is consistent with previous literature^71^.

Two additional proteins (GRK5, LRP4) are indirectly involved in the coagulation process. GRK5 is a member of the G protein-coupled receptor kinases, which have been previously suggested as potential drug targets for cardiovascular diseases^72,73^. GRK5 is involved in the regulation of thrombin-activated signaling in endothelial cells and platelets^74,75^, which is consistent with our finding of GRK5 associated with platelet markers. Similarly, LRP4 may participate in thrombotic cerebrovascular events by acting as a receptor of von Willebrand Factor (vWF), which is a key protein in the extrinsic coagulation pathway facilitating the binding of platelets to the injured vessel wall^76–78^. Targeting the LRP4–vWF complex has been suggested as a potential drug target for arterial wall remodeling^76^. Also, *LRP4* is highly expressed in brain tissue, particularly in astrocytes and oligodendrocytes, and there is evidence that LRP4 is critical to maintain the function of the neuromuscular junction^79^. Therefore, inhibiting LRP4 could lead to severe neurological adverse effects, as antibodies against LRP4 have been associated with myasthenia gravis and other myasthenia syndromes^79,80^.

Consistent with previous MR studies, we found that higher genetically-predicted plasma levels of MMP12 is related to lower risk of ischemic stroke^6,7^. MMP12 is involved in multiple biological functions^81^. In the context of ischemic stroke, a plausible mechanism supporting a protective role of MMP12 involves the regulation of coagulation through the cleavage of fibrinogen, a key glycoprotein that is converted into fibrin during clot formation, thereby reducing the substrate availability for thrombus formation^82^. In support of this hypothesis, evidence from animal models showed that knockout of *MMP12* leads to higher plasma levels of fibrinogen^83^.

Regulation of systolic blood pressure is another important pathway involved into the pathogenesis of ischemic stroke^10^, and our study showed that higher plasma levels of FURIN were associated with both higher risk of ischemic stroke and systolic blood pressure. *FURIN* is widely expressed across multiple tissues and regulates multiple physiological functions, including vascular remodeling, progression of atherosclerotic lesions, blood clotting system and the complement system^84–86^. This is consistent with our phenome-wide MR linking FURIN to circulating lipids, platelet markers, and liver and kidney function. However, mouse models indicate *FURIN* knockout is linked to developmental abnormalities, potentially limiting its suitability as a drug target for women of reproductive age^87^.

Our study offers insights into the potential role of energy metabolism and tricarboxylic acid cycle in the development of ischemic stroke through MMUT (methylmalonyl-CoA mutase), which is a vitamin B_12_-dependent mitochondrial enzyme, catalyzing the isomerisation of methylmalonyl-CoA to succinyl-CoA^88^. Genetic deficiency of MMUT leads to methylmalonic aciduria, a type of inborn error of metabolism, characterized by basal ganglia stroke^89,90^. Additionally, reduced activity of MMUT can be caused by vitamin B_12_ deficiency, which is consistent with our phenome-wide MR linking MMUT levels with markers of anemia due to B_12_ deficiency, including mean corpuscular volume and red blood cell distribution width. Therefore, disruption of the tricarboxylic acid cycle or brain hypo-perfusion due to anemia may explain the association of MMUT with ischemic stroke.

Our findings shed light into biological pathways through which CEP85 and SH3BGRL3 may contribute to the pathogenesis of ischemic stroke. Their genes are located in the same genetic locus, previously linked to platelet markers and LDL cholesterol^91,92^. Our phenome-wide MR further showed associations of CEP85 with platelet markers and lipids, and SH3BGRL3 with platelet markers and kidney function, pointing to potential pathways contributing to cerebrovascular pathology.

Our study showed an association between genetically-predicted SPATA20 and small vessel stroke, as well as associations with type 2 diabetes and systolic blood pressure, which are established risk factors for small vessel disease^93^. These findings offer insights into potential mechanistic pathways through which SPATA20 may contribute to the pathogenesis of small vessel stroke. Furthermore, the *SPATA20* locus has been associated with kidney function^94^, and our phenome-wide MR analysis confirmed this in the UK Biobank. Indeed, there is observational evidence linking impaired kidney function with small vessel disease^95^, and kidney impairment is the result of end organ damage due to both hypertension and type 2 diabetes.

In our study, we applied the MR approach, an instrumental variable technique with genetic variants serving as instruments for protein abundance to infer its causal effect on risk of ischemic stroke. MR relies on three core assumptions that should be satisfied by a genetic variant^96^. First, the genetic variants should be associated with protein abundance (*relevance assumption*). This assumption was satisfied by using a strict statistical significance threshold for variant selection and requiring an *F*-statistic greater than 10. Second, the genetic variants should be independent of any confounders of the association of protein abundance with risk of ischemic stroke (*independence assumption*). Although this assumption is not directly testable, we sought to minimize its violation by selecting only *cis* pQTLs, which are less prone to pleiotropy than *trans* pQTLs. In addition, we tested the genetic effects of the selected *cis* pQTLs on all the Olink and SomaScan proteins measured in the UK Biobank and deCODE study. Third, the genetic variants should affect risk of ischemic stroke only through the protein of interest and not through alternative pathways (*exclusion restriction assumption*). While this assumption is also untestable, we assessed its potential violation by performing colocalization analyses assuming a single causal variant at the *cis* region.

Currently, eight proteome-wide MR studies have investigated the role of plasma proteome in the risk of ischemic stroke and its subtypes. The majority of these studies used smaller proteogenomic datasets alongside the meta-analysis of GWAS data for ischemic stroke by the MEGASTROKE Consortium, which includes about 34,000 ischemic stroke cases of European ancestry. In our analysis, we confirmed the associations of GRK5, F11, MMP12, LRP4, PROCR, SH3BGRL3 and FURIN with ischemic stroke that were reported by the previous MR studies. In addition to this, we also provided genetic evidence supporting the association of MMUT, CEP85 and KNG1 with ischemic stroke, and the association of SPATA20 with small vessel stroke.

Our study leverages data on more proteomic markers, including comprehensively comparing data from two proteomic platforms and thereby providing greater discovery potential than in previously published work. In addition, we enhanced power and phenotypic depth by utilizing the largest available GWAS meta-analysis of ischemic stroke from the GIGASTROKE Consortium, including nearly twice as many ischemic stroke cases of European ancestry (62,100 cases) than previously available. We also provide a comprehensive framework of downstream analyses that offers additional insights into the biological evidence for the proteins identified, and we examined the effects of the stroke-associated proteins on established cardiovascular risk factors, using the largest and most up-to-date GWAS. Furthermore, we agnostically tested for potential additional effects of the stroke-associated proteins across the phenome, by performing a PheWAS in the UK Biobank and FinnGen, and conducting a comprehensive assessment of potential pleiotropic effects of the *cis* pQTLs by testing their effects on other plasma proteins in order to distinguish between occasions of mediated and confounded effects by capitalizing on biological information. This unique combination of data and downstream approaches offers meaningful biological insights and advances that have not been considered in previous proteome-wide MR studies of ischemic stroke.

Our study has several limitations. Firstly, although our study was the largest to date, we had limited power to identify plasma proteins that are specific to individual ischemic stroke subtypes. In addition, we were unable to examine plasma proteins that did not have available *cis* pQTLs. Third, MR estimates assume lifelong exposure to altered protein levels and therefore do not necessarily reflect the impact on a therapeutic intervention. Fourth, while we assessed pleiotropy by evaluating the association of *cis* pQTLs with other plasma proteins, pleiotropic effects on unmeasured proteins cannot be ruled out. Fifth, *cis* pQTLs were only available in European ancestry individuals, and further validation in other populations is needed. We were able to evaluate the associations of these *cis* pQTLs in the large-scale multi-ancestry GIGASTROKE dataset, as well as in Europeans only, and established directionally consistent causal effects. Identification of ancestry-specific causal proteins requires further study. Finally, despite integrating bioinformatics databases to distinguish between vertical and horizontal pleiotropy, our ability to do so is constrained by the current gaps in biological knowledge.

## Conclusion

Leveraging large-scale genetic and proteomic data, we identified 11 plasma proteins with likely causal relevance for ischemic stroke, including seven previously-identified proteins (GRK5, F11, PROCR, FURIN, LRP4, SH3BGRL3, and MMP12) and four proteins (MMUT, CEP85, KNG1, and SPATA20) that have not previously implicated in the risk of ischemic stroke. By integrating prior biological knowledge, we were able to show that these candidate proteins are involved in key physiological pathways, including coagulation, regulation of blood pressure and energy metabolism. Also, after considering potential undesirable effects and tissue specificity, CEP85, F11, GRK5, KNG1, and MMUT were the most promising candidates among those we considered for drug development aimed at ischemic stroke prevention. The continually expanding coverage of proteomic panels will facilitate the study of more plasma proteins and support development of new methods to harness *trans* pQTLs, as well as offer considerable future potential for further elucidating the role of proteins in ischemic stroke pathogenesis.

## Supplementary information

Transparent Peer Review file

Supplementary Information

Description of supplementary data files

Supplementary Data 1

Supplementary Data 2

Supplementary Data 3

Supplementary Data 4

Supplementary Data 5

Supplementary Data 6

Supplementary Data 7

Supplementary Data 8

Supplementary Data 9

Supplementary Data 10

Supplementary Data 11

Supplementary Data 12

Supplementary Data 13

## Data Availability

Source data for Fig. 2 can be found in Supplementary Data 2. Source data for Fig. 3 can be found in Supplementary Data 2 and 8. Source data for Fig. 4 can be found in Supplementary Data 8 and 9. All data used in the statistical analyses of this research study are publicly available. The *cis* pQTLs that were used as instrumental variables are publicly available in the relevant publications^8,9^. Specifically, pQTLs from the Olink platform in the UK Biobank are publicly available through Synapse (https://www.synapse.org/Synapse:syn51364943/wiki/622119), whereas pQTLs from the SomaScan platform in the deCODE Health Study are publicly available in the deCODE web portal (https://www.decode.com/summarydata/). GWAS summary statistics for ischemic stroke, ischemic stroke subtypes, and systolic blood pressure are publicly available through GWAS Catalog (https://www.ebi.ac.uk/gwas/), under the following accession numbers: GCST90104540 for any ischemic stroke, GCST90104542 for large artery stroke, GCST90104541 for cardioembolic stroke, GCST90104543 for small vessel stroke, and GCST90310294 for systolic blood pressure. GWAS summary statistics for ever smoking are publicly available through GWAS and Sequencing Consortium of Alcohol and Nicotine use (10.13020/przg-dp88). GWAS summary statistics for body mass index are publicly available through GIANT consortium (https://giant-consortium.web.broadinstitute.org/GIANT_consortium_data_files). GWAS summary statistics for type 2 diabetes are publicly available through DIAGRAM consortium (https://www.diagram-consortium.org/downloads.html). GWAS summary statistics for atrial fibrillation are publicly available through the Cardiovascular Knowledge Portal (https://cvd.hugeamp.org/downloads.html).

## Code availability

We used publicly available software for the statistical analyses in our study. Mendelian randomization analyses were performed using the TwoSampleMR package (https://mrcieu.github.io/TwoSampleMR/). Genetic colocalization analyses were conducted using the coloc package (https://github.com/chr1swallace/coloc). Linkage disequilibrium clumping was performed using the ieugwasr package (https://github.com/MRCIEU/ieugwasr) and PLINK version 1.90 (https://www.cog-genomics.org/plink/). Enrichment analysis was performed using the clusterProfiler package (https://github.com/YuLab-SMU/clusterProfiler).
