# Supplementary material for: Integrative proteogenomic analyses identify plasma proteins that impact the risk of ischemic stroke
Source: Commun Med (Lond). 2026 Jul 3;6:374. doi: 10.1038/s43856-026-01734-z (PMC13332204; doi:10.1038/s43856-026-01734-z)
Supplement: Supplementary file 2 — Supplementary Information [file 43856_2026_1734_MOESM2_ESM.pdf]

SUPPLEMENTARY MATERIAL

Integrative proteogenomic analyses identify plasma proteins that impact the risk of ischemic stroke

Lazaros Belbasis[1], Adam von Ende[1], Parag Gajendragadkar[1], Elsa Valdes-Marquez[1], Federico Murgia[1], Cornelia van Duijn[1], Jemma C Hopewell[1]

[1]Nuffield Department of Population Health, University of Oxford, Oxford, U.K.

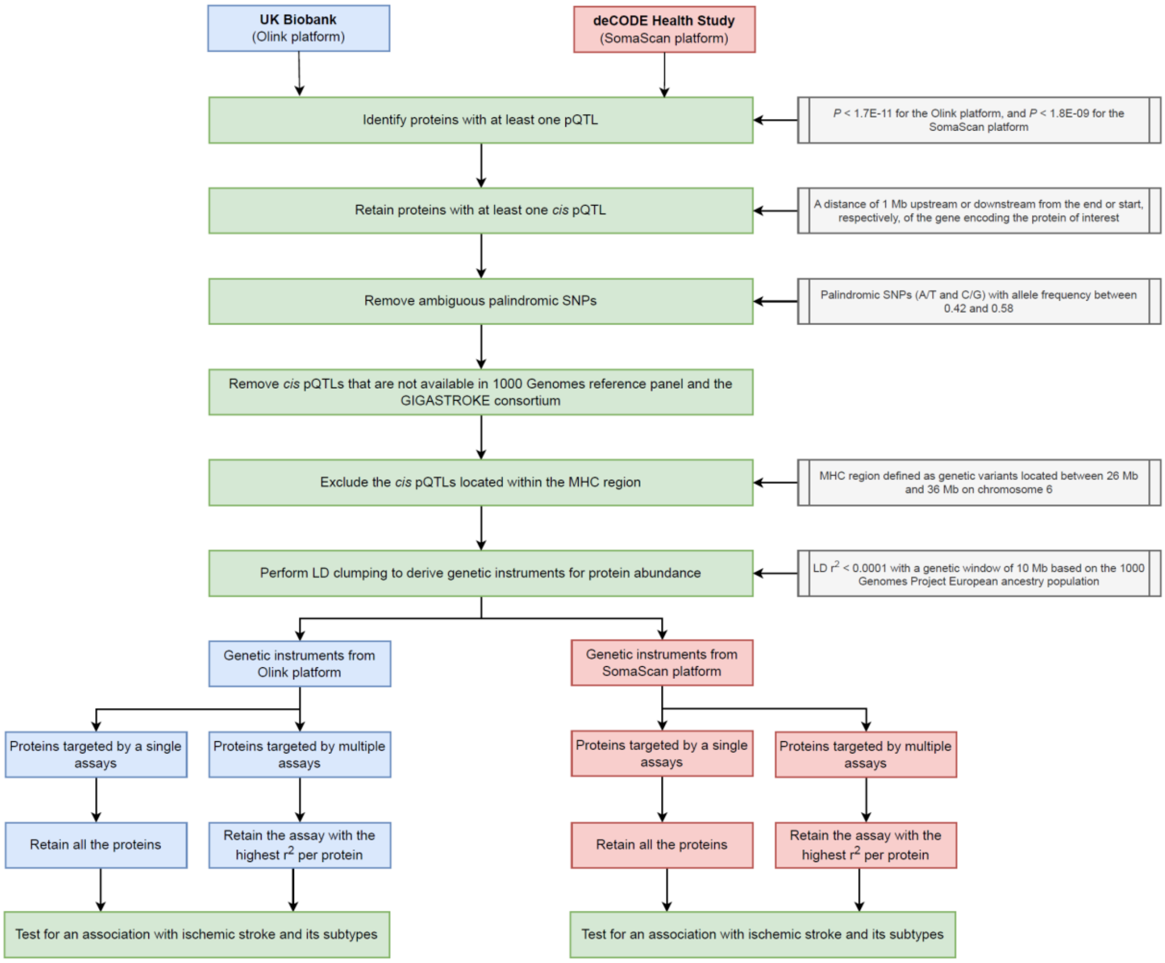

Supplementary Figure S1. Selection of genetic instruments for Mendelian randomization analysis.

Flow chart illustrating the systematic selection and quality control pipeline used to derive genetic instruments for protein abundance from the UK Biobank (Olink platform) and the deCODE Health Study (SomaScan platform). Proteins were first required to have at least one genome-wide significant pQTL and at least one *cis* pQTL, defined as variants within ±1 Mb of the gene encoding the protein. Ambiguous palindromic variants were excluded, as were *cis* pQTLs unavailable in the 1000Genomes reference panel or the GIGASTROKE consortium, and variants located within the major histocompatibility complex (MHC) region. Independent genetic instruments were then obtained through linkage disequilibrium (LD) clumping using European ancestry reference data. For proteins measured by multiple assays, the assay explaining the greatest proportion of variance (highest r^2) was retained. The resulting sets of genetic instruments were used to test associations with ischaemic stroke and its subtypes.

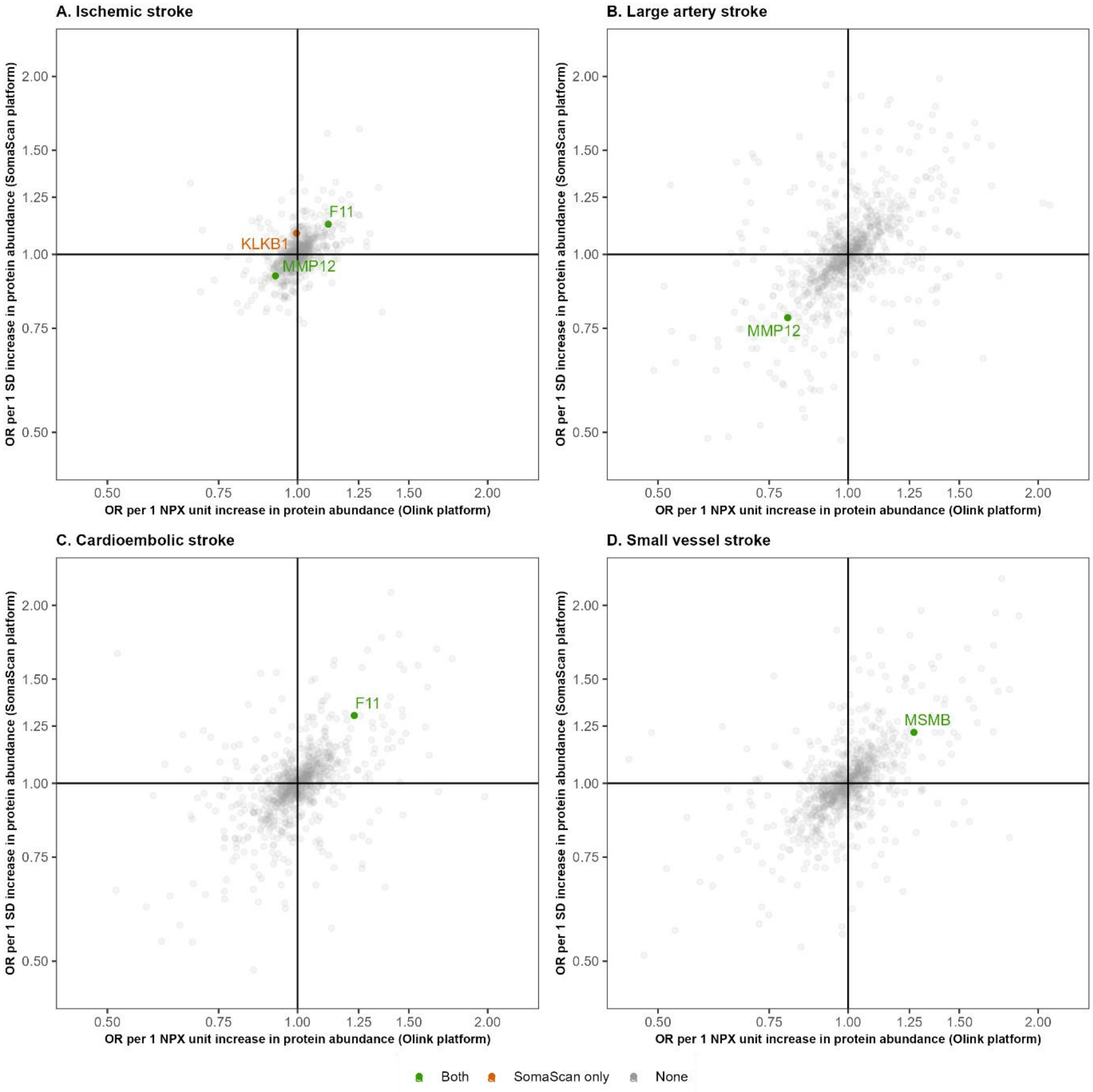

Supplementary Figure S2. MR effect estimate of plasma protein abundance on ischemic stroke and its subtypes based on two different proteomic platforms. The scatter plots allow for a comparison of findings across the two proteomic platforms for ischemic stroke (A) and its subtypes: large artery stroke (B), cardioembolic stroke (C), and small vessel stroke (D). The x-axis represents ORs using *cis* pQTLs from the Olink platform, while the y-axis represents ORs using *cis* pQTLs from the SomaScan platform. Proteins that were statistically significant using either *cis* pQTLs from either Olink or SomaScan are annotated. Proteins statistically significant in both platforms are labelled in green, proteins statistically significant only in the SomaScan platform are labelled in orange, and proteins that were not associated with ischemic stroke phenotypes are labelled in grey.

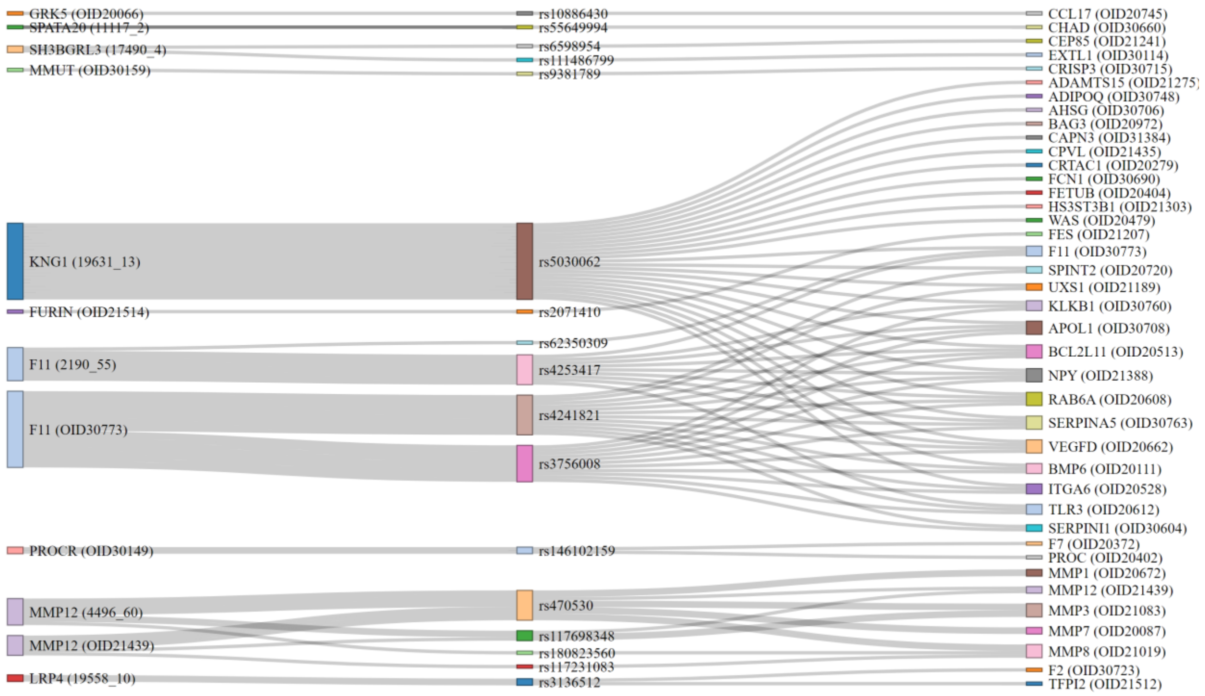

Supplementary Figure S3. Effect of genetic instrumental variables for the ischemic stroke-associated proteins on the abundance of plasma proteins measured through Olink platform.

The left column includes the proteins with a statistically significant effect on ischemic stroke and/or its subtypes. The middle column includes the *cis* pQTLs which (a) are used as instrumental variables for the proteins with a statistically significant association with ischemic stroke (or its subtypes) and (b) are also pQTLs for other proteins measured on the Olink platform. The right column shows these plasma proteins influenced by these *cis* pQTLs.

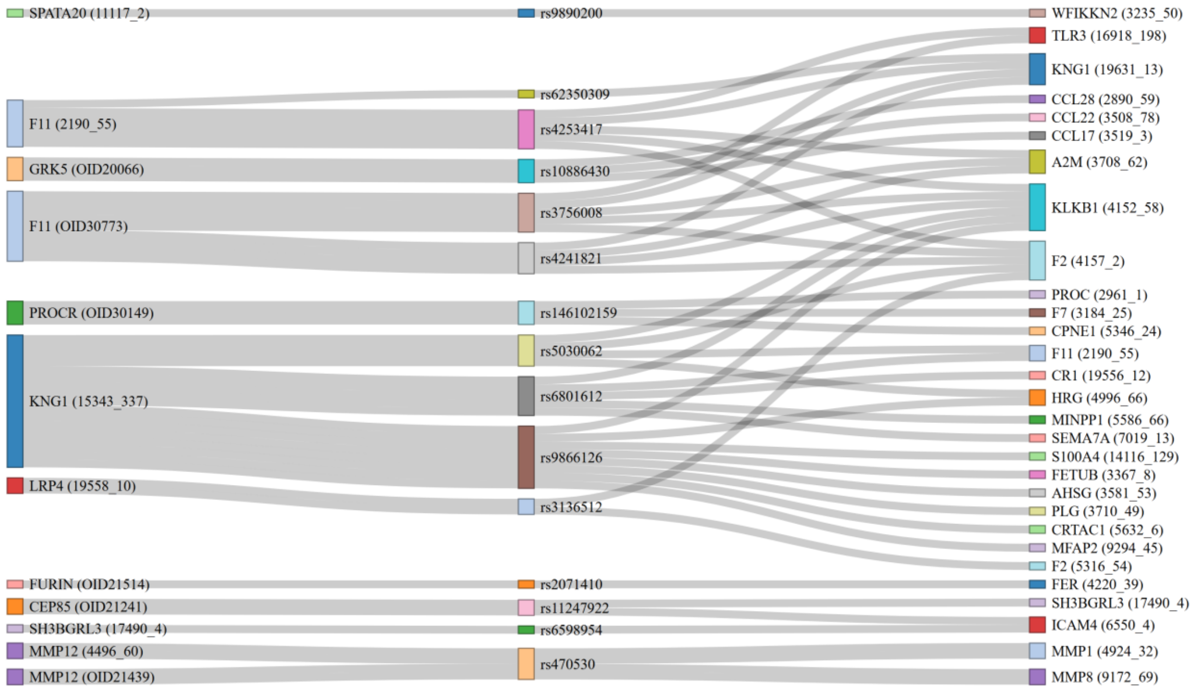

Supplementary Figure S4. Effect of genetic instrumental variables for the ischemic stroke-associated proteins on the abundance of plasma proteins measured through SomaScan platform.

The left column includes the proteins with a statistically significant effect on ischemic stroke and/or its subtypes. The middle column includes the *cis* pQTLs which (a) are used as instrumental variables for the proteins with a statistically significant association with ischemic stroke (or its subtypes) and (b) are also pQTLs for other proteins measured on the SomaScan platform. The right column shows these plasma proteins influenced by these *cis* pQTLs.

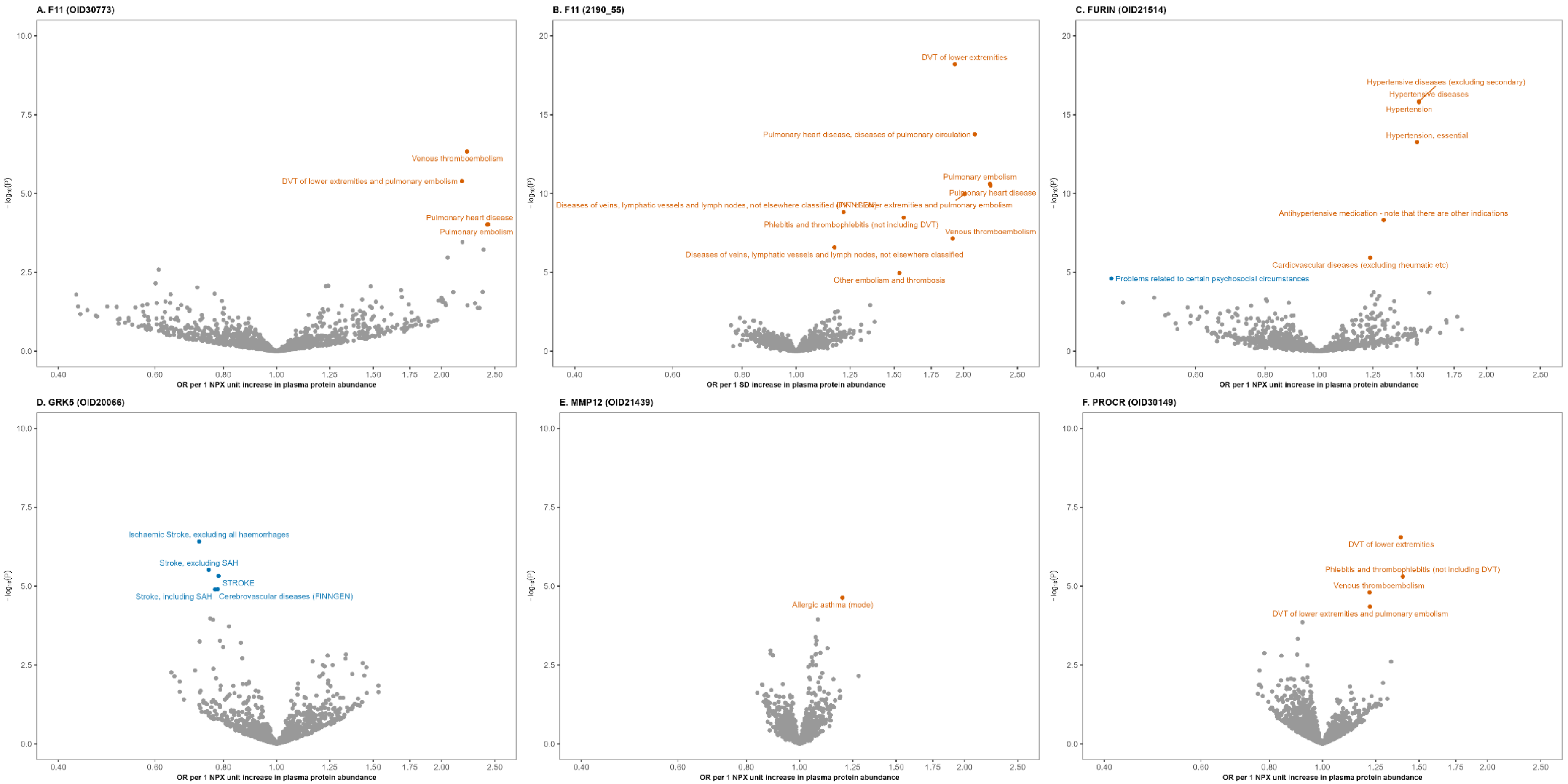

Supplementary Figure S5. Mendelian randomisation analysis for the effect of ischemic stroke-associated proteins on multiple health-related outcomes in FinnGen.

Each panel represents a different protein, showing only those with at least one statistically significant association with a health outcome. Statistically significant associations (at 5% FDR) are highlighted in orange (positive associations) and blue (negative associations). Grey dots indicate outcomes without statistically significant association.

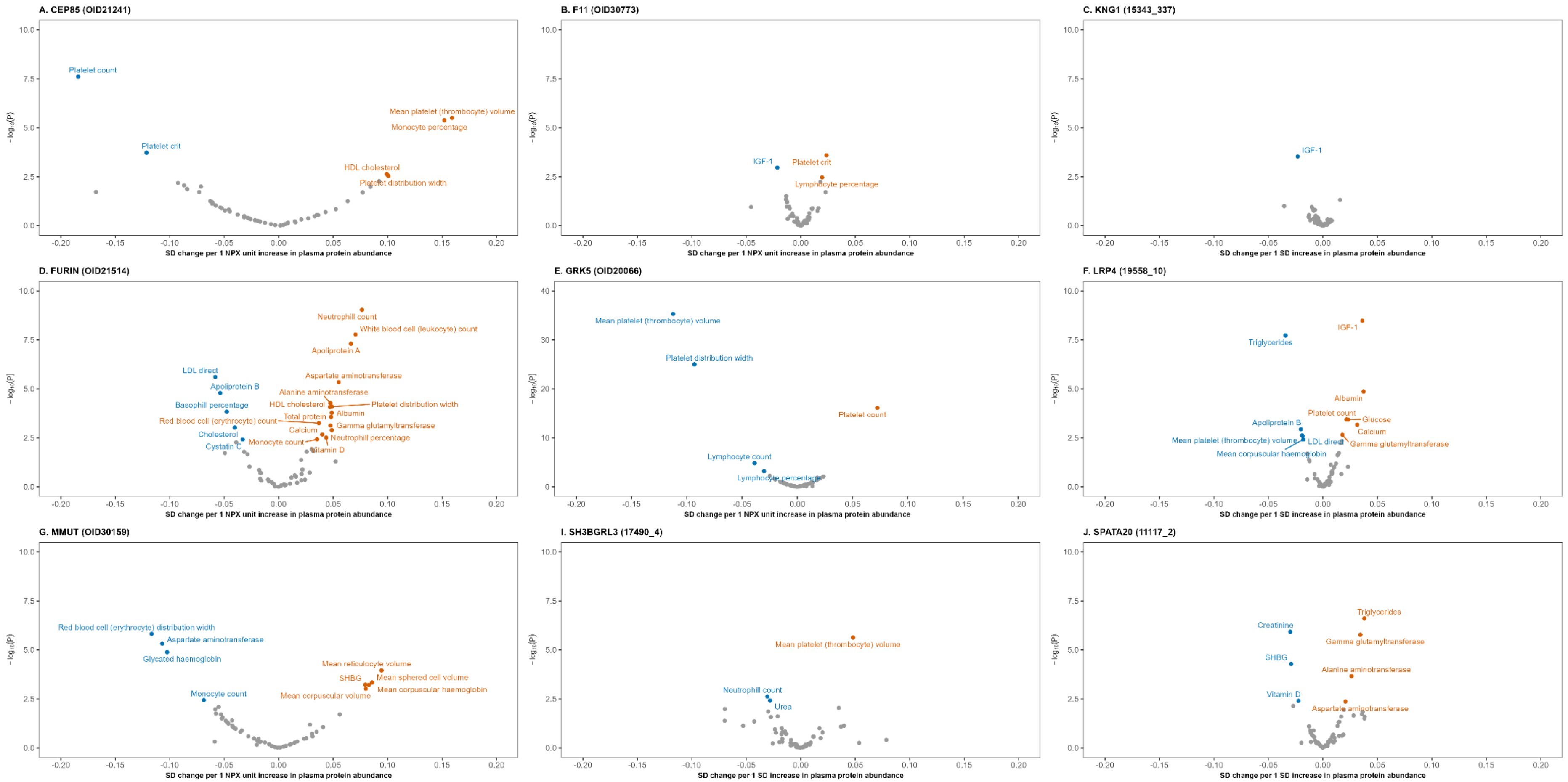

Supplementary Figure S6. Mendelian randomisation analysis for the association of ischemic stroke-associated proteins with blood biomarkers in the UK Biobank.

Each panel (A-J) represents a different protein, showing only those with at least one statistically significant association with a blood biomarker. Statistically significant associations are highlighted in blue for negative associations and orange for positive associations, while statistically non-significant associations are represented in grey.
