# Supplementary material for: Integrative proteogenomic analyses identify plasma proteins that impact the risk of ischemic stroke
Source: Commun Med (Lond). 2026 Jul 3;6:374. doi: 10.1038/s43856-026-01734-z (PMC13332204; doi:10.1038/s43856-026-01734-z)
Supplement: Supplementary file 3 — Description of supplementary data files [file 43856_2026_1734_MOESM3_ESM.docx]

**Description of Supplementary Data files**

**Supplementary Data 1.** Descriptive characteristics of the genome-wide association studies included in this study.

**Supplementary Data 2.** Associations of plasma proteins with any ischemic stroke and its subtypes estimated using a two-sample Mendelian randomization framework.

**Supplementary Data 3.** Associations of plasma proteins with any ischemic stroke estimated using the multi-ancestry GIGASTROKE dataset in a two-sample Mendelian randomization framework.

**Supplementary Data 4.** Colocalization analyses between plasma protein abundance and ischemic stroke phenotypes under the assumption of a single shared causal variant.

**Supplementary Data 5.** Sensitivity analysis for plasma proteins associated with ischemic stroke or its subtypes in the main analysis using the MR-link-2 method.

**Supplementary Data 6.** Comparison of effect estimates for ischemic stroke phenotypes derived from plasma proteins measured using both the Olink and SomaScan platforms.

**Supplementary Data 7.** Reverse Mendelian randomization analyses assessing the causal effects of genetic liability to ischemic stroke on plasma proteins.

**Supplementary Data 8.** Genetic effects of the cis pQTLs of stroke-associated proteins on other proteins measured in Olink platform.

**Supplementary Data 9.** Genetic effects of the cis pQTLs of stroke-associated proteins on other proteins measured in SomaScan platform.

**Supplementary Data 10.** Associations of plasma proteins with established ischemic stroke risk factors estimated using a two-sample Mendelian randomization framework.

**Supplementary Data 11.** Colocalization analyses between plasma protein abundance and established ischemic stroke risk factors under the assumption of a single shared causal variant.

**Supplementary Data 12.** Phenome-wide Mendelian randomization analysis conducted in FinnGen.

**Supplementary Data 13.** Phenome-wide Mendelian randomization analysis conducted in the UK Biobank.
