# Supplementary material for: Integrative proteogenomic analyses identify plasma proteins that impact the risk of ischemic stroke
Source: Commun Med (Lond). 2026 Jul 3;6:374. doi: 10.1038/s43856-026-01734-z (PMC13332204; doi:10.1038/s43856-026-01734-z)
Supplement: Supplementary file 1 — Transparent Peer Review file [file 43856_2026_1734_MOESM1_ESM.pdf]

nature portfolio

Peer Review File

Integrative proteogenomic analyses identify plasma proteins that impact the risk of ischemic stroke

Corresponding Author: Dr Lazaros Belbasis

This manuscript has been previously reviewed at another journal. This document only contains information relating to versions considered at Communications Medicine.

This file contains all reviewer reports in order by version, followed by all author rebuttals in order by version.

Version 0:

Reviewer comments:

Reviewer #1

(Remarks to the Author)
I reviewed a previous version of this manuscript, and I would like to commend the authors for their clear and thoughtful answers to the reviewer's comments and to their revisions of the manuscript. I believe that these responses and revisions adequately address the key points raised by myself and the other reviewers. I therefore do not have any further comments.

Reviewer #2

(Remarks to the Author)
I appreciate the authors' efforts to revise the manuscript. However, I still have one minor comment. Reviewer 1 suggested the investigation of potential pleiotropy. Please also consider using newly developed cis-MR methods, e.g. MR-link2.

Open Access This Peer Review File is licensed under a Creative Commons Attribution 4.0 International License, which permits use, sharing, adaptation, distribution and reproduction in any medium or format, as long as you give appropriate credit to the original author(s) and the source, provide a link to the Creative Commons license, and indicate if changes were made.
In cases where reviewers are anonymous, credit should be given to 'Anonymous Referee' and the source.
The images or other third party material in this Peer Review File are included in the article's Creative Commons license, unless indicated otherwise in a credit line to the material. If material is not included in the article's Creative Commons license and your intended use is not permitted by statutory regulation or exceeds the permitted use, you will need to obtain permission directly from the copyright holder.
To view a copy of this license, visit https://creativecommons.org/licenses/by/4.0/

Manuscript ID: COMMSMED-25-2513-T

Manuscript title: Integrative proteogenomic analyses discover novel plasma proteins that impact risk of ischemic stroke

Response to reviewers' comments

Reviewers' comments:

Reviewer #1 (Remarks to the Author):

I reviewed a previous version of this manuscript, and I would like to commend the authors for their clear and thoughtful answers to the reviewer's comments and to their revisions of the manuscript. I believe that these responses and revisions adequately address the key points raised by myself and the other reviewers. I therefore do not have any further comments.

Response

We would like to thank the reviewer for their positive comments.

Reviewer #2 (Remarks to the Author):

I appreciate the authors' efforts to revise the manuscript. However, I still have one minor comment. Reviewer 1 suggested the investigation of potential pleiotropy. Please also consider using newly developed cis-MR methods, e.g. MR-link2.

Response

In our revised manuscript, following reviewer's recommendation, we applied the MR-link-2 method. We have described the method as follows (page 11):

> "*As a sensitivity analysis, we applied the MR-link-2 method to proteins that showed evidence of a causal effect in the primary analysis. MR-link-2 is a pleiotropy-robust cis MR method that enables causal inference in the presence of LD and unobserved pleiotropic effects, without requiring the removal of pleiotropic genetic instruments or explicit measurement of all sources of pleiotropy. Using this approach, we evaluated the robustness of our findings to potential horizontal pleiotropy (i.e., violation of exclusion restriction assumption). MR-link-2 tests two parameters: the causal effect of a protein on the outcome conditional on pleiotropic effects, and a pleiotropy parameter that captures the heritable component of the outcome within the cis protein locus that is independent of the protein. For these analyses, we used summary statistics for each protein and stroke outcome across the corresponding cis region, together with the LD reference panel from the 1000 Genomes Project European ancestry population.*"

Out of 21 associations tested using MR-link-2, 19 associations remained statistically significant and all the effects were directionally consistent with the main analysis. We reported the results of MR-link-2 as follows (page 17):

> "*Results were broadly consistent in the sensitivity analysis using MR-link-2, a pleiotropy-robust cis MR method.22 Of the 21 associations detected in our primary analysis, 19 (90%) remained statistically significant after adjusting for pleiotropy (P <0.05; Supplementary Table S5). For SH2B3, the pleiotropy-adjusted effect on risk of ischemic stroke was not statistically significant, with the MR-link-2 pleiotropy test indicating substantial horizontal pleiotropy. This is consistent with the lack of colocalization between SH2B3 and ischemic stroke at the cis locus, as we previously reported. For MMUT, the pleiotropy-adjusted effect estimate was directionally consistent with the primary analysis but was not statistically significant. This finding may reflect a combination of limited statistical power due to low heritability for MMUT at the cis region and presence of horizontal pleiotropy.*"

We further report the full results of the MR-link-2 analyses in the Supplementary Table S5.
